# Surveillance of compliance with tobacco regulations in Örebro County, Sweden: a mixed methods study after the ban of test purchases

**DOI:** 10.1186/1747-597X-7-8

**Published:** 2012-02-15

**Authors:** Lisa Schölin, Charli Eriksson

**Affiliations:** 1 School of Health and Medical Sciences, Örebro University, S-701 82 Örebro, Sweden

**Keywords:** tobacco prevention, surveillance, tobacco legislation, thematic surveillance, mixed methods

## Abstract

**Background:**

Tobacco has long been known to be one of the most common reasons for sickness and premature deaths in the world. An important aspect of tobacco use is the youth's access to tobacco, and surveillance visits are one way to make sure how retailers are complying with age limit in the tobacco law. In Örebro County, Sweden, a project to reinforce the tobacco legislation was carried out in 2009-2010. One part of the project was surveillance visits that were done according to three different themes, called thematic surveillance.

**Methods:**

This study is an evaluation of the results from thematic surveillance and has a mixed methods approach. The quantitative analyses concerns protocols from 217 surveillance visits, where questions were asked about three themes (self-monitoring programs; marketing; labeling of products and pricing). In addition, questionnaires filled out by six tobacco administrators who worked within the project were analyzed qualitatively by content analysis in order to study their perceptions and opinions of the project.

**Results:**

This study shows that half of the visited retailers had self-monitoring programs. Lack of self-monitoring programs was significantly more common in smaller stores/kiosks and at restaurants. Further, the tobacco administrators who worked within the project perceived thematic surveillance as a good method for accomplishing better structure in surveillance work, but not as effective as purchase attempts (mystery shopping).

**Conclusions:**

Thematic surveillance was perceived as positive and the method was also regarded to be a good way to work with surveillance. However, the method could be developed further for optimal use and better effect at the retailers. It is clear that people who work with tobacco prevention at the local level in Örebro County want to use purchase attempts as a surveillance method, and that they believe that purchase attempts is the best way to make sure if store comply with the tobacco law.

## Background

Tobacco use, in particular cigarette smoking, is one of the main causes for illness and premature deaths in the world [1-3]. The prevalence of smoking in Sweden is according to the National Board of Health and Welfare (NBHW) 15% among women and 12% among men, in the population age 16 and older. Daily smoking is most common in the age group 45-64 years where 21% of the women and 17% of the men smokes on daily basis [4]. Research has shown that onset of smoking is most common in youth even though smoking among youths in Sweden is less common today as compared to the 1970s; there has been an increase of smoking among youths in ninth grade in compulsory school the last years. In 2009 the proportion of girls smoking on daily basis was 14% and 8% of the boys in 2^nd ^grade of upper secondary school. Even though smoking is more common among girls than boys, the tobacco consumption is almost equal when snuff use is included [4].

The overall goal for the tobacco politics in Sweden is to decrease the tobacco consumption, and one of the specific goals is to decrease the number of youths under 18 years that start smoking by 50% by 2014 [5]. This study focuses tobacco on prevention at the local level through an evaluation of a project to reinforce tobacco surveillance in Örebro County. This project was a part of a national effort to work harder to achieve the main goal with the tobacco politics by 2014, and is an important way to prevent youths from getting access to tobacco. Having access to tobacco is one contributing factor for whether youths will smoke or not [6], which makes it important to discover the sources where youths get cigarettes from.

Access to tobacco is an important factor for whether or not youths will smoke [6]. Even though minimum-age laws exist in many countries, minors still manage to buy tobacco. In several countries in Europe, studies have showed that youths seem be able to get access to tobacco through commercial sources, that is that they can buy cigarettes even though a minimum-age law exists on buying cigarettes [7-9]

Gallus et al [7] studied the perceived accessibility of tobacco for minors in Italy. Italian people ≥ 15 years were asked about their perceptions of the tobacco-sales law. Regarding perceived ease for minors to buy tobacco, 91.2% could not recall that they had ever seen a minor being refused to buy tobacco. Even though this study focused on perceptions and not actual sales rates, it gives some perspective of how easy it might be for Italian minors to get access to tobacco products. According to Rossow, Pedersen and Lund [8] similar conditions seem to exist among Norwegian youths. A school survey of pupils aged 13-17 showed that about two fifths of the youths who were tobacco users had bought their own tobacco. Out of the youths who smoked 40% reported that they bought their own cigarettes and 45% of the snuff users bought snuff by themselves. Sund and Hagqvist [9] studied perceived accessibility to tobacco with respect to the implementation of a minimum-age law in 1997. The study showed a decrease of having bought tobacco in shops from year 1996 to 2000, but more than 70% of the youths in all grades had bought tobacco on their own in 2000. More recent research shows that 40% of the boys and 35% of the girls in 9^th ^grade in compulsory school buy tobacco themselves. In 2^nd ^grade of upper secondary school, 67% of the boys and 52% of the girls buy their own tobacco [10].

Several studies have focused on investigating whether or not youths can buy tobacco in shops, and also addressed the issue of what variables that can predict or explain successful purchase attempts. Arday et al [11] concluded that no demographic variables in their purchase study were significant for the success of the purchase attempt. However, presence of warning signs and being asked for ID were significant for affecting the sales attempt. When the costumer was asked for ID, no purchase attempts succeeded. In addition, Rossow, Pedersen and Lund [8] showed that the proportion of minors that had bought their own tobacco was higher among those who reported being daily tobacco users. There was also a higher proportion that bought their own tobacco among those who perceived themselves as looking older than peers equal in age.

### Tobacco Legislation

#### The policy perspective on tobacco use among youths

An important perspective of tobacco legislation and tobacco sales to minors is the effectiveness of restrictions, such as sales bans and minimum-age laws. Several studies from different countries have shown that the proportion of youths who buy tobacco in shops without being of the legal age to buy cigarettes, decrease with respect to implementation of minimum-age laws [9,12,13]. However, the effectiveness of such laws can be discussed. In Finland, the implementation of sales bans resulted in significant decreases of daily smoking youths who bought their own tobacco. The smoking prevalence also decreased after the implementation of the sales bans. A significant decrease was evident among 14- and 16-year olds from 2001 to 2003 [12]. The California STAKE act was implemented in California, USA in 1995 and aimed at reducing the youth's access to tobacco. Purchase attempts were carried out in 1994 before the act had been implemented, in 1995 when the implementation took place, and then after the implementation in 1996, 1998 and 1999. A significant decrease in successful attempts was evident and implied that 16-year olds were 3-4 times more likely to get to purchase cigarettes before the STAKE act was implemented than after. The conclusion is that the enforcement of the act had an effect on the possibility for youths to purchase tobacco [13]. Even though implementation of laws or restrictions on tobacco sales, there is a risk that decreases in youths who buy cigarettes from stores, i.e. commercial sources, might lead to a transition towards getting access to tobacco through social sources. Instead of trying to buy tobacco in shops, youths instead tend to start buying tobacco from older friends or family members [9,12]. This implies that there is a broader perspective on accessibility to tobacco; however the focus is how policy implementation and restrictions are of importance for preventing youths from getting access to tobacco.

#### The Swedish tobacco law

The Swedish tobacco legislation includes several different parts which all aim at decreasing the negative effects due to tobacco use. The Swedish tobacco law covers smoke free environments, smoke free work places, warning signs and labeling of tobacco products, restrictions about tobacco sales and import, marketing and product checks. This section will only concern the parts of the Swedish tobacco law that includes restrictions on tobacco sales [14].

The development of the Swedish tobacco law began in 1995 with a suggestion of legislation regarding purchase of tobacco. The government bill 1995/96:228 regarded this issue which was followed by the implementation of a minimum-age law in 1997, where it became illegal to sell tobacco to people under 18. It became evident though that the law was not complied enough, which called for further restrictions. The government bill 2001/02:64 suggested interventions which were supposed to strengthen the surveillance. The bill included an obligation for all retailers to report selling tobacco, but also that municipalities could charge retailers for surveillance visits. Another important update of the legislation was done in 2003 when Sweden signed the Framework Convention on Tobacco Control. The framework is a minimum standard for legislation, but makes it possible for member countries to make stricter legislation. The government bill 2004/05:118 declared that all tobacco retailers became responsible to put up signs of the 18-year limit for purchasing tobacco and also to perform age checks on all types of tobacco sales. Furthermore, the bill also made clear that all tobacco retailers are required to have a self-monitoring program (NIPH, 2009). A self-monitoring program means that the retailer should have a document that clarifies the routines for complying with the tobacco law. This document is a way for the owner of the sales place, and its staff, to know what routines that is supposed to be followed when selling tobacco. The tobacco retailer is required to send a written program to the municipality that states the routines for complying with the tobacco law, and a copy of that should also be available at the sales place. The self-monitoring program should be a guide and provide support for the staff working at the place, and the routines that the sales place has should be well established and discussed with the staff. The main purpose of self-monitoring programs is support to the staff at the sales place, so that they know how they should act in certain situations. It is also an important feature to emphasize the responsibility of the person who is selling tobacco [15].

#### Responsibility for the surveillance on national, regional and local level

The overall aim with the tobacco policies in Sweden is to reduce the harm caused by tobacco use. Interventions within the area of tobacco prevention have a broad approach and include different types of actions, like legislation and information. Preventing youths and children from initiating smoking is one of the most central objectives with the Swedish tobacco legislation, and is the base for all tobacco interventions. The Swedish National Institute of Public Health (NIPH) is the central authority with responsibility for tobacco surveillance, for example surveillance on warning signs for tobacco products.

The County Administrative Boards in Sweden have the responsibility to monitor the work of the municipalities, but they also have the task to promote cooperation between several authorities that are involved in the work of tobacco law enforcement. Finally, the municipalities are responsible for the direct surveillance of compliance of the tobacco law [14].

### The project - Thematic Surveillance in Örebro County

#### Background

Thematic surveillance in Örebro County was a project within a national effort to reinforce tobacco surveillance in all of Sweden. The reason for this was that the Swedish Government perceived that the tobacco legislation was not complied enough, and that improvements had to be made to stop youths from getting access to tobacco. In 2008, NIPH received the mission from the government to make further efforts in tobacco prevention for three years. This intervention was called "The Tobacco Mission 2008-2010" and had the purpose to contribute to achieving the goals for the tobacco politics in Sweden by 2014. A reason for the national investment in tobacco prevention was that the government perceived powerful legislation as one of the most important features for the Swedish alcohol, drug, doping, and tobacco politics [16].

The County Administrative Boards every year receive a letter of regulation from the Swedish Government, which describes what the Councils are supposed to work with the following year. The tasks within the tobacco area in 2009 were to report to NIPH how the County Administrative Boards had been worked with surveillance, according to the tobacco law. The boards were also bounded to report what efforts had been made within the municipalities and the results of such efforts [[17] p.21]. The regulation letter in 2010 once again expressed that the County Administrative Boards should report how the surveillance work had been performed and the actions taken to strengthen the local surveillance work. Further, the Government stated that the surveillance work should be developed and coordinated at the regional level [[18] p.21].

#### Structure

The project in question was a regional campaign within the Tobacco Mission 2008-2010. The overall aim with the project was to reinforce tobacco legislation and thematic surveillance was a part of the two-year project, implemented in the second year of the project. The first year, 2009, focused on purchase attempts that were performed in collaboration with the municipalities in Örebro County [19]. However, the same year a decision was made by the Parliamentary Ombudsmen to ban municipalities to use purchase attempts (mystery shopping) in surveillance work. The reason why this method could no longer be used regarding both tobacco and middle strength beer was according to the Parliamentary Ombudsmen that the method was unconventional exercise of a public authority [20].

The surveillance visits that were performed during 2010 were done according to three central themes; 1) self-monitoring programs, 2) marketing and, 3) labeling of products and pricing. These three themes were phrased as concrete questions and were the base for the surveillance protocols. The surveillance visits were classified according to how many themes that were investigated at the visit. One theme was considered as low level of surveillance, two themes as medium and all check of all three themes were classified as high level of surveillance.

Central for the thematic surveillance project was to visit tobacco retailers according to the themes described above. For the surveillance visits it was required that the municipalities checked all of the three themes and that it was supposed to be done in order with the highest surveillance level [19].

#### Aims

Thematic surveillance was a part of a bigger campaign with focus on reinforced tobacco legislation to accomplish the national goals for the tobacco politics by 2014. The aims of this study are to evaluate the project and highlight the positive experiences, but also parts that need to be developed and strengthened in the future. For this purpose the study has a mixed methods approach. The overall aim is to evaluate how tobacco retailers in the county have implemented existing requirements of tobacco legislation, due to the results from the surveillance visits. The aim is also to study how the thematic surveillance project was perceived by people who worked with the project in the municipalities. The central research questions for this study are;

1. What are the general tendencies among the tobacco retailers, based on the themes of the surveillance visits?

2. What geographical differences are there between the municipalities?

○ What variables predict the lack of self-monitoring programs?

3. What are the experiences from the tobacco administrators that worked within the project?

4. What are the perceived strengths and weaknesses of the project?

## Method

### Design of the evaluation

The method section consists of two parts; due to the research design of this evaluation. Since a mixed methods approach has been used to analyze the results from the project, separate sections are needed for the quantitative and qualitative method. Mixed methods research is grounded in the pragmatic research tradition which emphasizes that the research question is the most central factor for choosing methodology. The mixed methods approach in research allows the researcher to work from both an explaining and descriptive perspective, in a combination, instead of choosing one single method [21].

### Thematic surveillance - the Quantitative Approach

#### Sample

In total, protocols from 217 surveillance visits were received from the County Administrative Board. The municipalities range in population size from less than 6000 (Laxå) to 135 000 (Örebro) inhabitants. The sample of places to visit for surveillance was all gas stations and smaller shops in the county, and the 35 places that sold tobacco to minors in the purchase attempts in 2009. This sample was a minimum recommendation from the County Administrative Board of Örebro, and the municipalities could add places according to preference and knowledge about the stores. Many municipalities chose to do more visits than the recommendation [19]. Protocols from 217 surveillance visits within thematic surveillance have been analyzed.

#### Measures

During the project, surveillance visits in Örebro County were carried out using a protocol developed in collaboration between the County Administrative Board of Örebro and the County Administrative Board of Värmland. This protocol included 23 items was used to check all three themes (self-monitoring programs; marketing; labeling of products and pricing). The person who performed the visit asked the question and wrote down the answers in the protocol.

#### Procedure

The surveillance visits were carried out by the tobacco administrators and other people who work within the municipality. The tobacco administrators got information and training in how to fill out the protocol in the beginning of 2010. In the store setting, they presented themselves to the retailer and the purpose of their visit, and then asked the questions in the protocol. The document had a space where the person who performed the visit was supposed to put their name, but not all protocol was handed in with a name, which made it difficult to know how many different persons had performed the visits.

#### Statistical analyses of the surveillance protocols

The statistical analysis is based on the results from the surveillance protocols. In this analysis only the first theme in the protocol (self-monitoring programs and tobacco stickers) has been analyzed, with respect to the relevance of the evaluation. Marketing and prizing was not relevant for the purpose of the evaluation. Significance testing was performed by using Pearson's chi-square test [22]. Differences between variables were compared with the chi-square test, where statistical significance was tested at < .05 *P*-value.

Logistic regression analysis was performed on the dichotomous variable having self-monitoring program (dependent variable) and a set of independent variables (tobacco stickers at the counter, municipality, type of sales place, age for ID-checks, and if many youths try to buy tobacco). The analysis tested the predictability of the categorical outcomes on the model, and was run in SPSS by the forward conditional method.

### Qualitative Approach to experiences of Thematic Surveillance

#### Sample

In the project reinforced tobacco surveillance in Örebro County, there were representative tobacco administrators in all of the municipalities in the county. The project was carried out in 10 of the 12 municipalities in the county; two did not perform surveillance visits according to the thematic surveillance project. One of the administrators is responsible for five municipalities, and the others have one municipality each, so the total numbers of administrators that participated in the project was six. One of these administrators was a woman and the other five were men. The administrators ranged in age from 23 to about 60 years of age. Years of working with surveillance ranged from about one year to 24 years. All of the administrators had some type of university degree. One of non-attending municipalities was using a protocol that they had developed themselves and structured their surveillance work according to this protocol. The other municipality reported that surveillance had not been performed according to thematic surveillance due to sickness. The evaluation of the perceptions of the project therefore included 10 of the 12 municipalities in the county, but all participating municipalities. The sample for the qualitative data collection was all of the tobacco administrators in the municipalities who had the main responsibility for the tobacco surveillance, including thematic surveillance. However, other people than the administrators performed surveillance, but this study only focused on the six persons who had the main responsibility for tobacco surveillance in their municipalities.

#### Measures and procedure

An email questionnaire with questions divided into four different sections were created and sent to the six tobacco administrators. The sections were the surveillance, the project, the future and some personal questions. The administrators received an email where the evaluation was explained in short and instructions about how to answer and return the questionnaire. The questionnaire itself contained a letter with further descriptions about the evaluation and why the administrator's answers were important. The administrators were told in the introduction letter in the questionnaire that participation was voluntary, but that an important part of the evaluation was about the experiences of the coordinators which made their participation important for the evaluation. The administrators were also informed that their responses would be coded, so that their names would not appear in relation to their responses.

#### Qualitative analysis of the questionnaires

**Figure 1:**
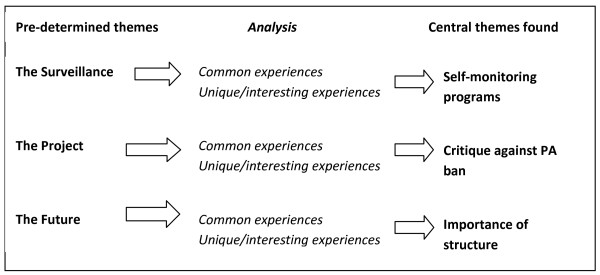
**The qualitative analysis process**.

The analysis of the responses was done by exploring different themes in the answers, which was done stepwise. The questionnaires already had categories (surveillance, project, future and personal) which was the base for the analysis. According to the concept of content analysis, the questionnaires were summarized with all the answers put in one document. From the pre-determined themes, the answers were analyzed based on common experiences and unique/interesting experiences. The process is visualized in Figure 1.

The answers to the questions in the questionnaires were given in Swedish, and so when quoting the respondents the quotes had to be translated. This has been done so that the English quote is as close to the Swedish as possible. Although, some quotes had to be revised to make sense in English but this should not be an issue since the quotes are still expressing the same idea.

## Results

### Quantitative Analysis of the Thematic Surveillance Visits

#### Descriptive statistics of the protocols

The quantitative analysis concerns the surveillance protocols (*N *= 217) that were used during the surveillance visits within the project thematic surveillance. The number of visits per municipality is described in Table 1 and shows that Örebro, which is the largest municipality, had the highest number of surveillance visits.

**Table 1:** Overview of the surveillance visits

	Number of visits (%)
Askersund	15 (6.9)
Degerfors	5 (2.3)
Hallsberg	24 (11.1)
Karlskoga	26 (12.0)
Kumla	18 (8.3)
Laxå	7 (3.2)
Lekeberg	8 (3.7)
Lindesberg	14 (6.5)
Nora	10 (4.6)
Örebro	90 (41.5)

Total	217 (100)

**Figure 2:**
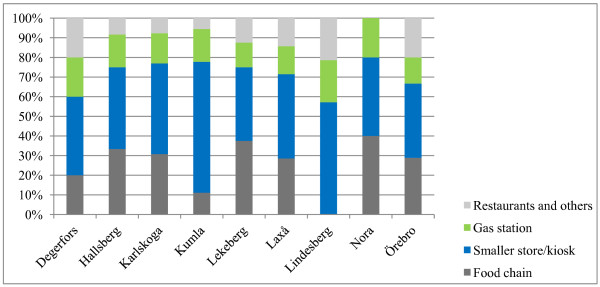
**Type of sales places in the municipalities**.

The sales places that were visited within thematic surveillance were divided into four categories; grocery chains, smaller stores (i.e. not grocery chains) and kiosks, gas stations, and restaurants and other types of sales places (Figure 2). There were differences over the municipalities regarding proportions of the different types of sales places. A higher proportion of grocery chains were apparent in, for example Lekeberg, while none of the visited places in Lindesberg was a grocery chain.

#### Youths trying to buy tobacco and ID checks

The question was if many youths try to buy tobacco in the current store and 76 places (35.5%) reported that many youths try to buy tobacco and 138 places (64.5%) did not perceive that many youths try to buy tobacco in that store. On the question if costumers are asked for ID, all of the 213 places that had answered that question reported that costumers are being asked for ID.

The age for when ID is checked varied between the sales places. At 76 of the sales places (37.6%) the response was that ID is checked for people perceived to be less than 25 years old. The second most common response, at 39 of the places (19.3%), was that ID is checked when the clerk feels suspicious about a costumer's age. At 27 sales places (13.4%), ID was always checked for everyone; 19 places (9.4%) reported other reasons such as if the customer is unknown or if the customer looks childish; 15 places (7.4%) check for ID at 18 years of age; 12 places (5.9%) check under 30 years of age; 8 places (4.0%) check under 20 and 6 places (3%) responded that they check for ID if the customer look young. The age for ID checks were not significant when tested against type of sales place, having tobacco stickers at the counter, or whether many youths try to buy tobacco in the current store.

#### Presence of self-monitoring programs

Overall, 102 places (50%) that were visited had self-monitoring programs and the rest did not have self-monitoring programs. In Hallsberg, Karlskoga and Örebro the majority of the sales places reported that they did not have self-monitoring programs. The differences in proportion of places with self monitoring programs depending on the municipality was significant (*χ*^2^(9) = 41.078; p < .001), which means that stores in for example Lekeberg is more likely to have self-monitoring programs than stores in for example Karlskoga.

There was a significant difference between grocery chains and gas stations (also chains), and smaller grocery stores and kiosks as well as restaurants and other places. Presence of self-monitoring programs is therefore associated with the type of sales place (Table 2). Grocery stores and gas stations are more likely to have self-monitoring programs, while smaller stores/kiosks and restaurants are more likely to not have self-monitoring programs.

**Table 2:** Frequency of self-monitoring programs according to type of sales place, percentage in parenthesis

	Have self-monitoring program		
	Yes (%)	No (%)	Total
Grocery chains	48 (81.4)	11 (18.6)	59
Smaller store/kiosk	25 (29.1)	61 (70.9)	86
Gas station	21 (70.0)	9 (30.0)	30
Restaurants and others	8 (27.6)	21 (72.4)	29

	102	102	204

χ^2^(3) = 48.901; p < .001

Another question is if the appearance of the store is related to having a self-monitoring program. In this case, the appearance is defined as if the stores have tobacco stickers at the counter. A significant association was found between the appearance in the store setting, regarding tobacco stickers, and having a self monitoring program (Table 3).

**Table 3:** Frequency of self-monitoring programs and presence of tobacco stickers at the counter, percentage in parenthesis

	Have self-monitoring program		
Have stickers	Yes (%)	No (%)	Total
Yes	76 (76.8)	63 (23.2)	139
No	23 (61.8)	39 (38.2)	62

	99	102	201

χ ^2^(1) = 5.031; p <.05

#### Updates of self-monitoring programs and occasion for informing the staff

At 22 places (36.7%) information about the self-monitoring program was at new employment, 22. The second most common occasion was at staff meetings, where 13 places (21.7%) informed their employees about the self monitoring programs. Among the less frequent answers were that the staff was informed once or twice a year at staff meeting and new employment or that information was given continuously.

#### Presence of tobacco stickers

The majority of the sales places had tobacco stickers at the counter. In total, 146 sales places (69.2%) had stickers at the counter, while 65 places (30.8%) did not have stickers. When looking at the presence of stickers according to sales place it is evident that smaller stores and kiosks, restaurants and others in less extent had stickers at the counter than grocery chain and gas stations (Table 4).

**Table 4:** Presence of tobacco stickers at the counter according to type of sales place

	Have tobacco stickers		
	
	Yes (%)	No (%)	Total (%)
Grocery chain	44 (77.2)	13 (22.8)	57 (100)
Smaller store/kiosk	67 (72.8)	25 (27.2)	92 (100)
Gas station	22 (68.8)	10 (31.3)	32 (100)
Restaurants and others	13 (43.2)	17 (56.7)	30 (100)
	146	65	211

χ ^2^(3 =)11.696; p < .01

#### What predicts lack of self-monitoring programs?

To analyze which variables predict lack of self-monitoring programs logistic regression analysis was used to see which variables that did fit the model. The dependent variable in this analysis was having self-monitoring program (yes or no) and following independent variables were included in the model: presence of tobacco sticker at the counter; municipality; sales place; age for ID-checks; many youths try to buy tobacco. Logistic regression was carried out using the stepwise conditional method, and the full model classified 82.7% of the cases correctly. The analysis showed that the independent variables contributing to predicting lack of self-monitoring programs were type of sales place and municipality (Table 5). Both independent variables that were significant in the model had a reference category which was the base for the analysis. The reference category for type of sales place was grocery chains, because this was the largest group in this category. Örebro served as reference category for municipality, since it is the largest municipality in the region. The logistic regression shows that smaller kiosks and restaurants were significant predictors to lack of self-monitoring programs in the first step of the analysis, and also in the second step. The other contributing variable in the model was municipality, where Karlskoga was a significant predictor. This means that visited stores in Karlskoga was more likely to be missing self-monitoring programs.

**Table 5:** Logistic regression for lack of self monitoring program

		Adjusted	CI (95%)	
			
Variable	p-value	OR	Lower	Upper
Smaller kiosks	.000	41.225	12.617	134.703
Gas stations	.29	4.010	1.155	13.922
Restaurants and others	.000	45.397	9.946	207.217
Degerfors	.768	1.628	.064	41.316
Karlskoga	.009	7.295	1.651	32.239
Hallsberg	.881	1.172	.320	4.288
Askersund	.001	.049	.008	.289
Kumla	.012	.149	.034	.656
Lindesberg	.000	.016	.002	.144
Laxå	.008	.042	.004	.439
Lekeberg	.999	.000	.000	-
Nora	.037	.118	.016	.883

Cox & Snell R Square = .444; Nagelkerke R Square = .592

### Thematic Surveillance - Qualitative Analysis

#### Experiences from the project and perceptions about the thematic surveillance method

The common experiences from the surveillance visits focused on the thematic surveillance as a method. Before the project started, several of the administrators reported that the surveillance work mainly included purchase attempts. One important part of the method thematic surveillance that was expressed by several of the administrators was the focus on self-monitoring programs. This was good according to one administrator, since it was "more focused on specific areas" (P2). Moreover, it was also perceived that the method provided better contact with the retailers;

"The dialog, possibility to inform, the mapping of different kinds of tobacco, pricing etc." (P5)

Another important aspect was that it was positive that the same surveillance method had been used in almost all the municipalities in the county. One tobacco administrator expressed that the focus on the self-monitoring programs made stores more or less forced to get self-monitoring programs. The administrator perceived this as positive, since the stores that did not have self-monitoring programs then had to get one and also to learn what it was all about. The overall experiences from the thematic surveillance visits were good, one wrote that this method was better because it was "more thorough and structured" (P1). Some of the overall experiences from the thematic surveillance visits were;

"Good, but a percentage of the retailers think that the municipality should use recourses to chase the real sinners instead of them." (P1)

Another thought about the experiences from thematic surveillance was that the method improved the contact between the municipality and retailers, because of further contact with the places that did not have self-monitoring programs. In addition, another administrator expressed that "it has been a good guide" (P6).

Nevertheless, not everything about the thematic surveillance visits was perceived as positive. Things that were perceived as less positive with this surveillance method was that it "takes more time" (P1) and that the focus on certain themes might have resulted in that "other parts of the surveillance might have been neglected" (P4). Another negative perception about the method was that the surveillance visits did not focus on tobacco that had incorrect labeling or if the stores sold single cigarettes. One tobacco administrator reported the experiences of thematic surveillance as;

"The surveillance visits felt more like 'information visits' than surveillance visits. There was inadequate information about what you should do if the business did not behave" (P3)

#### Contact with the County Administrative Board and perceptions about the project

All of the tobacco administrators reported that they had received the information about the project from the County Administrative Board. Some specified that the information was given at a meeting with a representative from the County Administrative Board. The experiences were good regarding support during the project and the tobacco administrators were positive about their contact with the County Administrative Board and perceived the contact as helpful in the surveillance work. One perceived the support from the Board as;

"Good, positive feedback from the administrator at the County Administrative Board. And in collaboration with colleagues within the area" (P2)

There were different perceptions from the administrators of what the project had meant for their work. One thought that it had provided better overview over the sales places and another perceived that it had given more structure to the surveillance work. It was also perceived that it was "a way to make sure that store kept their self-monitoring programs updated" (P2). When asked "what does reinforced surveillance mean to you?" many responses regarded purchase attempts, which had been the main way of working with surveillances earlier. One also thought that it was a way to see other circumstances that could not be detected by purchase attempts.

Another aspect was that thematic surveillance offered a possibility to focus the surveillance work and make sure that places that did not live up to the demands for selling tobacco could be punished in some way.

"That you spend more time at surveillance and that you perform sanctions at business that are not behaving" (P3)

The overall experiences of the project differed. Some of the administrators just reported that they had positive experiences from the project and being positive for the future surveillance, without specifying what in particular they perceived as positive. Other noted that it was important to be two persons at the surveillance visits, not more than two and not less than two. It was also perceived that the project was making it a little more complicated for the unserious stores, which was perceived as positive;

"It is good that something is done to at least make life a little more complicated for unserious sales places, and also give serious a pat on the shoulder for good behavior" (P4)

#### The loss of purchase attempts as surveillance method and future surveillance work

In general, the tobacco administrators were very negative to the directives about not being able to use purchase attempts (mystery shopping) as a surveillance method. One noted that it would be good if it could be used as a complement to thematic surveillance. Another administrator expressed that not being able to use purchase attempts means losing the control about getting knowledge about if the retailers really are complying with the tobacco law. One tobacco administrator wrote that the new directives make the work more complicated since the police needs to be involved in this matter. The same person wrote that it can be hard to get help from the police since they have many other tasks, which makes these kinds of issues less of a priority. One of the administrators expressed the view of the directives of not being able to use purchase attempts as following;

"When JO (the Parliamentary Ombudsman) put the foot down in this case about a year ago it was like the air went out of us surveillance people. The work with purchase attempts in south Sweden had come to a point where we waited on the sentence in cases that presupposed from this work, when JO's announcement dropped down as a bomb. The new direction on surveillance during fall 2010 has a little of the character "what the hell do we do now". But we have to do something and what we do is still not bad" (P4)

The directives about not being able to use purchase attempts was perceived to affect the possibility to effective surveillance and also that it is necessary to use more resources to be able to accomplish the same results as purchase attempts. For the future surveillance work, three of the tobacco administrators reported that they will continue to work according to the thematic surveillance method. One of the administrators wrote that the future surveillance work will be done according to a model developed in Malmö, and was perceived to work really well. Experiences that the respondents will bring to future surveillance work were different. Among the responses it was evident that a structured form of document was important for working with surveillance. Experiences that one of the tobacco administrators will use for future surveillance work were as follows;

"We have developed a check list that we use at surveillance and leave on place. This is to decrease the administrative work. We also have routines for what to do when stores are not behaving" (P3)

Working together with other authorities, such as the police, was also reported as a way that will be a part of the future surveillance work. Focusing on some central themes over a time period was reported as a way to put extra focus on parts of the surveillance to make the work more intense. Although, what was also expressed from this view was that it is important to make sure that no other parts of the surveillance is neglected due to focusing on a central theme. The concept of checking each store in the same way was expressed as a good way to get knowledge about how tobacco is sold.

## Discussion

This study has evaluated the project thematic surveillance in Örebro County. A mixed methods approached have been used to get knowledge about how the project was carried out and also the results from the surveillance visits. The discussion section will address central issues of the findings, but also attempt to draw some conclusions of the study and give recommendations for the future surveillance work by a SWOT-analysis.

One thing that was perceived as positive with thematic surveillance was the use of the same method in all municipalities. The importance of using a protocol that is working well and make proper documentation of the sales places were also perceived as important for the surveillance work and was regarded as important for the future surveillance work. There were differences in how the protocols were filled out, which either can depend on how it was filled out or how the retailer answered the questions. The answer, or the formulation of the answer, from the person at the sales place are valuable to see, since it in some way express their knowledge about preventing youths from getting access to tobacco. Even so, improvements in the protocol might be possible so that there could be a better accuracy in completing the protocols. Since the perceptions among some of the tobacco administrators seem to be that they think it is important to investigate every store in the same way further improvements of the protocols maybe could enhance the structure of the visits.

One way to work for improvement with the protocols would be to have a discussion, with all administrators involved, where important experiences from the surveillance visits could be addressed. In that discussion the aim would be to agree on how the protocols should be filled out in the best possible way. By having a similar structure in all municipalities better accuracy could be accomplished which makes it easier to evaluate or compare different municipalities. This also means that the administrators should educate other people within the municipality that perform surveillance visits.

The quantitative analysis shows that the majority of the stores ask for ID when the costumer is under 25 years. Since the municipalities no longer can check whether or not, or when, the sales places ask for ID, nothing can be said about the validity of that statement. Critique against banning purchase attempts was expressed from all tobacco administrators and the perception was that surveillance will not be entirely effective if age checks cannot be examined by performing purchase attempts. All sales places reported that they ask costumers for ID, although there were differences regarding at what age ID was asked for. However, when looking at smoking prevalence in a local youth survey it can be concluded that youth in the county smoke, even though they don't have the age to buy cigarettes. For instance, in Askersund more than 15% of boys in 9^th ^grade are smoking cigarettes daily [23]. This is the case in other municipalities as well, meaning that some minors do get hold of cigarettes in some way. It cannot be concluded though where the youths get hold of the cigarettes. And what seems to be the only way to know the extent of accessibility to tobacco for the youths in the county is to do purchase attempts.

The directive to ban surveillance workers from using purchase attempts as a surveillance method was an issue that received a lot of the comments. All of the tobacco administrators were more or less expressing the same thoughts about the ban, that surveillance will never be completely effective if purchase attempts cannot be done. Several studies have showed that a minimum age-law is effective over time on youth's ability to buy tobacco in stores [9,12,13], but the question is how the tobacco legislation can be monitored without using methods that examines the reality at places that sell tobacco. On a higher level, this should be an important viewpoint for policy makers. Since the tobacco administrators in this evaluation express that they have been deprived their ability to really know which stores that are selling to minors, in other words not complying with the law, and that more resources have to be used to accomplish the same level of knowledge. It is important to listen to the opinions from the people who are working with surveillance at the local level, in order to be able to develop the surveillance work. If tobacco administrators feel like they have lost an important tool to work with surveillance, it creates a need to discuss if the directives can be changed in some way. As one of the tobacco administrators expressed it, more effort and resources must be used in order to accomplish the same results as with purchase attempts. According to Blanke and da Costa e Silva [24] legislation is "the heart of an effective program" when it comes to tobacco control. Moreover, appropriate data collection is important for effective policies of tobacco control programs. For example, "monitoring and evaluation the effects of legislation and developing improvements to strengthen policies" is important issues for capacity building within the work of tobacco control [24]. A Cochrane Review of 48 studies noted that giving retailers information was less effective in reducing illegal sales than active enforcement or multi-component educational strategies [25]. However, no strategy achieved complete sustained compliance. The need for collecting data of the effectiveness of legislation is therefore not only something that is important for the people working with prevention at the local level, but also expressed through the World Health Organization (WHO) as one of the important parts of capacity building in tobacco control.

The Swedish government presented the annual work plan for the alcohol, narcotics, doping and tobacco politics for 2011 in May 2011. In this plan, the government acknowledges that the surveillance work needs to be reinforced and one of the goals for 2011 is to investigate the possibility for municipalities to use purchase attempts as a surveillance method [26]. It is positive that the Government has paid attention to the issue of not being able to use purchase attempts as a surveillance method. The suggestion for further work with tobacco surveillance in Örebro County is to keep working with thematic surveillance and perhaps develop parts that can be improved, such as how to fill out the protocol. The most important part, considered by the evaluator, is to use the experiences of the tobacco administrators and discuss parts that are working and parts that are not working with this method. It has already been emphasized that it is positive to work in the same way in all 10 municipalities and this experience is important to for the further development of the method. Another important issue is to involve the non-participating. Improving the method should include finding out how the non-participating have worked with surveillance so that experiences and knowledge from these parts of the county can be included in the future work to. Finally, this research report is a contribution to the future surveillance work in Örebro County. Given the results from this evaluation it is easier to take the next step to reinforce the surveillance work so that fewer youths get access to tobacco, which will benefit the public health in the long run. Moreover, this study also gives argument for making the municipalities able to use purchase attempts as a surveillance method.

The question about self-monitoring programs is rather central in this evaluation. It seems that surveillance before the project did not focus on the presence of self-monitoring program, which can be an explanation to why a lot of the answers treated this question. Still, it was expressed that it is good to make sure that the self-monitoring programs are updated, but that it is not really possible to make sure that the store is working according to it, as supposed to. The only method for that is, according to several of the tobacco administrators, to do purchase attempts to get a fair picture of reality. According to NIPH [14], all tobacco retailers are required to have self-monitoring programs. Nevertheless it seems as if this is something that is often neglected by the retailers, or what might be more of an issue; lack of knowledge. The NIPH have developed material regarding tobacco legislation in several different languages so that information can be provided to retailers that might not have Swedish as their first language. The issue of understanding between the municipalities and retailers has probably been addressed before by people working with tobacco, but the reason to why so many sales places don't have self-monitoring programs should be investigated further. One way to do that is to try to open up for an open discussion between the surveillance person and retailers, with the focus to figure out what the retailers perceive as difficult with the tobacco law.

A vital issue in research is the ethical perspective. It is therefore important to discuss how ethical considerations have been treated in this study. Within human and social science, the Swedish Research Council has set up four standards of ethics that researchers have to comply with. The demands to which the researcher needs to address in all the stages of the research are information, consent, confidentiality and use of research [27]. These demands have been addressed when planning for and conducting this research, and no ethical considerations have been violated.

A type of analysis that is common within evaluation is the SWOT-analysis (acronym for Strengths, Weaknesses, Opportunities and Threats). A SWOT-analysis focus on how the overall experiences from a project could be valuable for future work. The purpose of this analysis is to summarize experiences that can be brought into future projects or for development of the method.

The central areas in thematic surveillance have been summarized in table 6. Strengths with thematic surveillance is that it seemed to be well established in the municipalities and that the contact with the County Administrative Board was good and provided good support for the people who worked at the local level. Further, it was perceived as a good method and a structured way to do surveillance. Since almost all municipalities in the county used the same protocol and worked according to the same method, it was possible to compare results from different municipalities. From an evaluation point of view, this kind of documentation was crucial for the possibility to evaluate the overall results. In addition to the strengths, one important focus of the weaknesses of the project, or the method per se, was that other parts of surveillance might have been neglected. When implementing new methods, there is always a risk that the focus of the new method will make people forget about other important things. This might also be connected to the impression that tobacco administrators in the municipalities did not really perceive this method as the most optimal surveillance method. If one believes that the method that is used is not the best way to do surveillance that might not make the person a hundred percent motivated to use the method. Since it seems as if using both methods according to several administrators would be the best way to do surveillance that gives the best result, that combination might also create bigger motivation for the surveillance work.

**Table 6:** SWOT-analysis of thematic surveillance

**S**trengths	• Good contact and positive approach towards the County Administrative Board • A structured method that improved the focus on specific variables within surveillance, such as self-monitoring programs • The method meant that many municipalities in the county worked in the same way which was perceived as positive
**W**eaknesses	• Focus on some parts of the surveillance might have led to that other parts were neglected • Not an optimal way to see if the stores really complies with the law (only purchase attempts can give an honest picture)
**O**pportunities	• Develop the method and perhaps make revisions in the surveillance protocol so that better accuracy can be achieved
**T**hreats	• The feeling of losing an important tool for surveillance work since purchase attempts cannot longer be used

The opportunities and threats of the project are strongly connected to whether or not the municipalities can use purchase attempts. It would be a good idea to improve the method of thematic surveillance, as mentioned earlier, to have an open discussion about how the protocol should be used. Improvements of the method could also include making some sort of check list to follow at the surveillance visit, apart from the protocol. To perform the visits as similar as possible could also be a positive thing, since the view on surveillance would be similar in the whole county. Nonetheless, it is clear that purchase attempts are an extremely important way for the municipalities to make sure that sales places are complying with the law. Forster, Widome and Bernat [28] conclude that there are two ways for minors to get access to tobacco; trough commercial or social sources. The authors also states that tobacco sales to minors can be prevented, or at least decreased, by working actively to enforce minimum-age laws, such as the Synar Amendment. Furthermore, the evidence of whether enforcement of minimum-age laws reduces smoking prevalence is mixed. However, local prevention work such as thematic surveillance is not only about decreasing smoking prevalence. It is also a way to implement a proper way of working to make sure that stores are complying with the minimum-age law. What Forster, Widome and Bernat [28] describes though, is that enforcement of minimum-age laws within the Synar law should be done by what they call "compliance checks by underage decoys". Doing purchase attempts is clearly not something that is just a matter of whether or not it is an acceptable method to use in Sweden, it seems that it is internationally a method for doing checkups on tobacco sales places. From a research point of view, municipalities ability to use purchase attempts in surveillance work would not only be good to strengthen the surveillance work, but also a contribution to the research community since sales rates to minors could be compared between different countries.

This study has contributed in getting greater knowledge about local tobacco prevention work. There are several areas that could be investigated further in order to develop prevention work at the local level. This study investigated a certain method used in surveillance visits, but it was evident that purchase attempts are perceived as the most effective method by people who work with tobacco surveillance. Further research should focus on studying the relationship between how the stores express that they work to prevent minors from buying tobacco, for example asking for ID or having a self-monitoring program, and results from actual purchase attempts. If municipalities will be able to use purchase attempts again as a surveillance method, it will be possible for tobacco administrators to compare the surveillance visits with successfulness in purchase attempts. It might also be interesting and important to investigate how thematic surveillance was used in the county of Värmland. Comparing these two counties and study in what way they might differ and have similarities would be a good way to use the knowledge of the project. One thing that would be of interest is to find out whether the statistically significant differences found in Örebro, for example in lack of self-monitoring programs and sales place, is the same in Värmland. The knowledge gained from this work can be used both to develop the local surveillance work, but also to give incitements at the national level for what is good evidence based practice.

## Conclusions

The project of reinforced tobacco surveillance was perceived as positive and the method was also regarded to be a good way to work with surveillance. However, the method could be developed further for optimal use and better effect at the retailers. It is clear that people who work with tobacco prevention at the local level in Örebro County want to use purchase attempts as a surveillance method, and that they believe that purchase attempts is the best way to make sure if store comply with the tobacco law.

## Competing interests

The authors declare that they have no competing interests.

## Authors' contributions

LS was the main author of the manuscript and was involved in all aspects of the study. CE was involved in the design of the study and he provided scientific oversights and feedback throughout the development of the study and the manuscript. The authors read and approved the final manuscript.
